# Supplementary material for: Mobile barrier mechanisms for Na+-coupled symport in an MFS sugar transporter
Source: bioRxiv. 2023 Nov 25:2023.09.18.558283. Originally published 2023 Sep 18. Preprint. [Version 2] doi: 10.1101/2023.09.18.558283 (PMC10542114; doi:10.1101/2023.09.18.558283)
Supplement: Supplement 1 [file media-1.pdf]

Supplemental materials

Mobile barrier mechanisms for Na^+-coupled symport in an MFS transporter

Parameswaran Hariharan[1], Yuqi, Shi[2], Satoshi Katsube[1], Katleen Willibal[3], Nathan D. Burrows[4], Patrick Mitchell[4], Amirhossein Bakhtiiari[5], Samantha Stanfield[1], Els Pardon[3], H. Ronald Kaback[6#], Ruibin Liang[5], Jan Steyaert[3], Rosa Viner[2], and Lan Guan[1§]

[1]Department of Cell Physiology and Molecular Biophysics, Center for Membrane Protein Research, Texas Tech University Health Sciences Center, School of Medicine, Lubbock, TX 79424, USA
[2]Thermo Fisher Scientific, San Jose, CA 95134, USA
[3]VIB-VUB Center for Structural Biology, 1050 Brussel, Belgium
[4]Division of CryoEM and Bioimaging, Stanford Synchrotron Radiation Light Source, SLAC National Accelerator Laboratory, Menlo Park, CA 94025, USA
[5]Department of Chemistry and Biochemistry, Texas Tech University, Lubbock, TX 79409, USA
[6]Department of Physiology, University of California, Los Angeles, Los Angeles, CA, USA
[#]Deceased
[§]To whom correspondence should be addressed: **Lan Guan,** E-mail: Lan.Guan@ttuhsc.edu

Contents:

- **Supplemental Table**
  sTable 1 Cryo-EM data collection and structure determination statistics.
  sTable 2 HDX reaction and labeling details.
  sTable 3. *E. coli* strains and plasmids used in this study.

- **Supplemental Figures**
  sFig. 1. Hybrid Nb725_4 generated by CDR grafting.
  sFig. 2. Effects of substrate/ligand binding on the Nbs binding.
  sFig. 3. Nb effects on substrate/ligand binding.
  sFig. 4. CryoEM data process.
  sFig. 5. GSFSC resolution and 3dFSC.
  sFig. 6. Evaluation of map and models.
  sFig. 7. NabFab comparison.
  sFig. 8. Interactions of Nb725m_4 and $MelB_{St}$.
  sFig. 9. Complex of $MelB_{St}$ with Nb725 and $EIIA^{Glc}$.
  sFig. 10. MD simulations of the Na^+ binding at both inward- and outward-facing states.
  sFig. 11. Galactose-binding pocket in the outward-facing crystal structure [PDB ID 7L17].
  sFig. 12. Alignment.
  sFig. 13. Membrane topology.
  sFig. 14. Histograms of deuterium uptake time courses.

sTable 1. Cryo-EM data collection and structure determination statistics.

	MelB$_{St}$/Nb725m/NabFab	
**EMDB Accession Code**	EMD-41062	
**PDB Accession ID**	8T60	
	Non-tilted collection	Tilted collection
**Data Collection**		
Microscope	Krios-TEMBETA	Krios- TEMBETA
Voltage (kV)	300	300
Number of movies	14,094	8,716
Electron dose (e^-/Å^2)	50.00	50.00
Defocus range (μm)	−0.8 to -1.8	−0.8 to -1.8
Pixel size (Å)	0.86	0.86
Plate tilt angel (Degree)	/	30
**Data Processing**		
Initial number of particles	7,632,727	2,887,147
Combined final number of particles	203,876	
Symmetry imposed	C1	
Map resolution* (Å)	3.29	
B factor	101.4	
**Model Refinement**		
Chains	5	
Non-hydrogen atoms	7503	
Protein residues	978	
Mean B-factor		
Protein	97.35	
Na^+ ion	88.64	
RMS deviations		
Bond lengths (Å)	0.003	
Bond angles (°)	0.509	
MolProbity score	1.82	
Clash score	4.09	
Poor rotamers (%)	1.98	
Ramachandran plot		
Favored (%)	93.48	
Allowed (%)	6.52	
Outliers (%)	0.00	
Model Resolution (Å)†	3.2/3.3/3.5	

*Resolution determined by Fourier shell coefficient threshold of 0.143 for corrected masked map

† Resolution determined between the model and the resolved map by Fourier shell coefficient threshold of 0/0.143/0.5

sTable 2. HDX reaction and labeling details.

Samples measured	WT MelB$_{St}$	WT MelB$_{St}$ complexed with Nb725m
HX reaction buffer	25 mM Tris-HCl, pD 7.5, 150 mM NaCl, 10% Glycerol, and 0.01% DDM	
Reaction temperature (°C)	20	
HX time course (s)	0, 30, 300, 3000	
Number of peptides	153	
Sequence coverage by labeling	86 %	
Mean peptide length	9.3	
Average redundancy	2.9	
Replicates (technical)	3	
|ΔD| (Da)	0.3071	
Back exchange rate	Not appliable	

sTable 3. *E. coli* strains and plasmids used in this study

	Description	Reference
**Stains**		
E. coli DW2	*melA*$^{+}$ Δ*melB* Δ*lacZY*	59
E. coli XL1 Blue	*recA1 endA1 gyrA96 thi-1 hsdR17 supE44 relA1 lac [F' proAB lacIqZΔM15 Tn10 (Tetr)]*	Agilent Technologies
E. coli ArcticExpress (DE3)	F$^{–}$ *ompT hsdS*(rB$^{–}$ mB$^{–}$) *dcm*$^{+}$ Tetr gal λ(DE3) *endA* Hte [cpn10 cpn60 Gentr]	Agilent Technologies
E. coli DH5α *cyaA*$^{-}$	Δ*cyaA*	29
E. coli BL21(DE3) T7 express	*fhuA2 lacZ::T7 gene1 [lon] ompT gal sulA11 R(mcr-73::miniTn10*--Tet^{S}*)2 [dcm] R(zgb-210::Tn10*--Tet^{S}*) endA1 Δ(mcrC-mrr)114::IS10*	New England Biolabs.
E. coli BL21(DE3) C43	F$^{–}$ *ompT hsdSB (rB- mB-) gal dcm (DE3)*	60
E. coli BL21(DE3) pRIL	F$^{–}$ ompT hsdS(rB$^{–}$ mB$^{–}$) dcm^{+} Tet^{r} gal endA Hte [argU ileY leuW], Cam^{r}	Agilent Technologies
**Plasmids**		
pCS19	pQE60 derivative inserted with gene *lacI*^{q}; amp^{r}	69
pACYC	pACYC/FX-derived vector for control; no *ccdB* gene: cam^{r}	70
pCS19/FX	Expression vector with two SapI sites and *ccdB* gene for FX cloning; amp^{r}	70
pACYC/MelB$_{St}$	Expression vector for MelB$_{St}$ derived from pACYC/FX; cam^{r}	70
pCS19/X:T18/FX	Expression plasmid for expressing a target protein "X" with a C-terminal fusion with T18 fragment; two SapI sites and *ccdB* gene for FX cloning; amp^{r}	29
pCS19/T18	pCS19/X:T18/FX-derived vector for expressing T18 fragment; no *ccdB* gene.	29
pACYC/T25	pACYC/T25:X/FX-derived vector for expressing T25 fragment; no *ccdB* gene	29
pACYC/T25:MelB$_{St}$	Expression plasmid for the T25:MelB$_{St}$ hybrid derived from pACYC/T25:X/FX; cam^{r}	29
pCS19/Nb725:T18	Expression plasmid for Nb725:T18 hybrid derived from pCS19/X:T18/FX; amp^{r}	29
pCS19/Nb725_4:T18	Expression plasmid for Nb728:T18 hybrid derived from pCS19/X:T18/FX; amp^{r}	This study
pCS19/Nb725	Expression plasmid for Nb725 derived from pCS19/FX; amp^{r}	29
pCS19/Nb725_4	Expression plasmid for Nb725_4 derived from pCS19/FX; amp^{r}	This study
pET26b(+)	Construction of Nb725_4 expression plasmid, construction of anti-Fab Nb expression plasmid; replacement pelB leader sequence for periplasmic expression, Kan^{r}	Novagen (EMD Millipore)
pET26/Nb725_4	Inducible periplasmic expression plasmid used for Nb725_4 protein production, Kan^{r}	This study
pET26/Anti-Fab Nb	Inducible periplasmic expression plasmid used for Anti-Fab Nb protein production, Kan^{r}	This study
p7XC3H/Nb725	Inducible cytoplasmic expression plasmid used for Nb725 protein production, Kan^{r}	29

p7XNH3/EIIA^{Glc}	Inducible cytoplasmic expression plasmid used for *E. coli* EIIA^{Glc} protein production, Kan^r	33
pR2.2/NabFab	Expression and purification of NabFab, Amp^r	36
pMSP1E3D1	Expression and purification of MSP1E3D1, Kan^r	Addgene/20066
pRK792	Expression and purification of TEV protease, Amp^r	Addgene/8830

Primers

Labels	Applications	Oligonucleotides
MelB_Nb	pCS19/Nb725_4:T18 (This adds C-terminal ‘A’ and allow the C-terminal fusion)	Fwd: 5′- ATATATGCTCTTCTAGTCAACGTCAATTGGTAG -3′
		Rev: 5′- TATATAGCTCTTCATGCGCTGCTCACGGTCAC -3′
MelB_FX	pCS19:Nb725_4-CTH (This adds C-terminal ‘HHHHHH and truncated by a stop codon)	Fwd: 5′- ATATATGCTCTTCTAGTATGCAACGTCAATTGGTAG -3′
		Rev: 5′- TATATAGCTCTTCATGCTTAGTGGTGATGATGGTGGTGGCT GCTCACGGTCAC -3′
Nb-PelB-NdeI	Add restriction site for Nb725_4 and Anti-Fab Nb construction	Fwd: 5’-TTTAAGAAGGAGATATACATATG-3’
Nb-STRP-XhoI	Add restriction site for Nb725_4 and Anti-Fab Nb construction	Rev: 5’TTTGTTCTAGACTCGAGTTATTTCTC-5’

Supplementary figures

sFig. 1

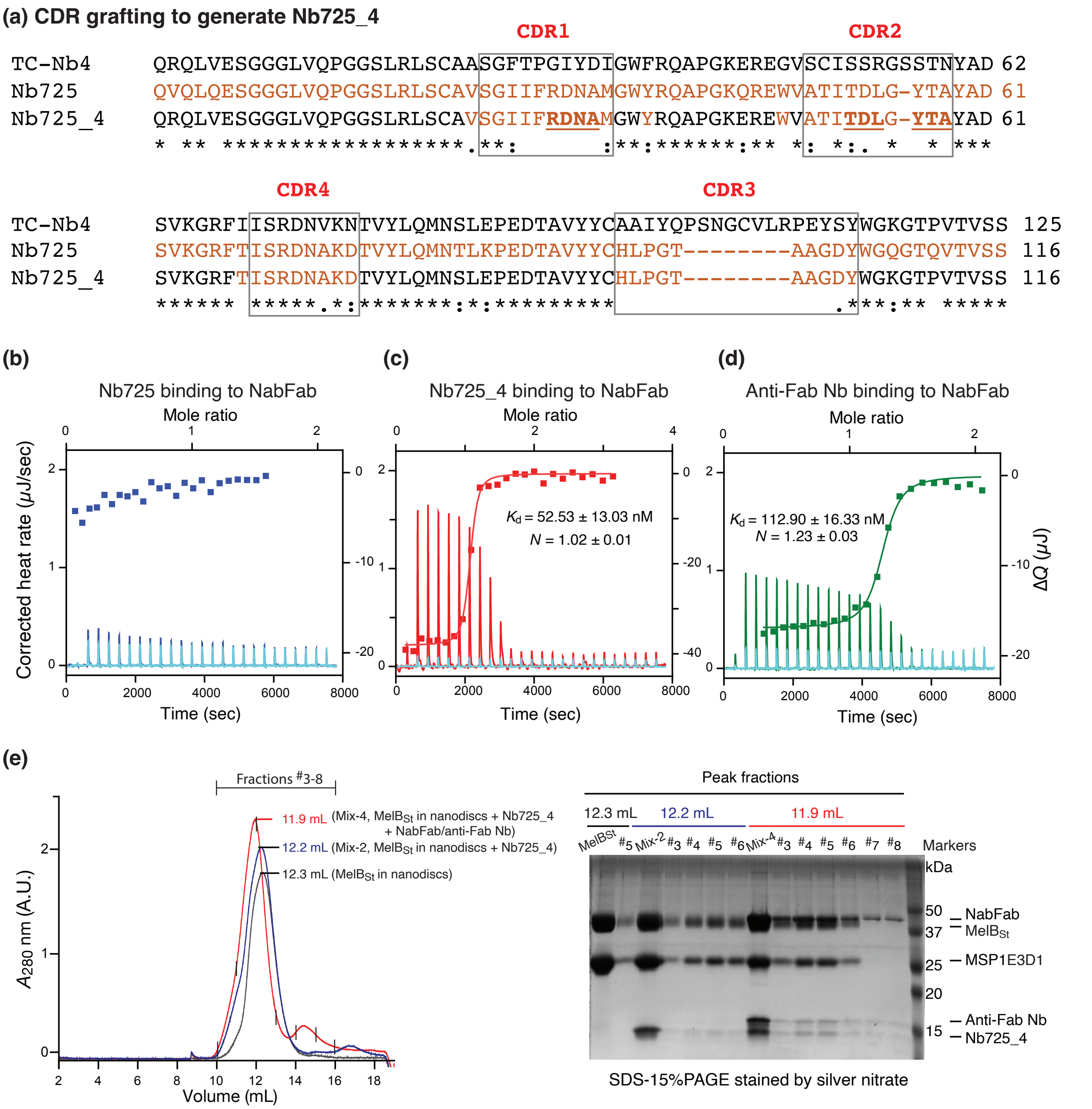

sFig. 1. Hybrid Nb725_4 generated by CDR grafting. (a) All CDR regions are indicated by boxes. The sequences of TC-Nb4 and MelB$_{St}$ Nb725 were colored in black and red, respectively. The sequence of hybrid Nb725_4 was colored in black and red from the two parents Nbs. The Nb725_4 binding residues were highlighted in underlined bold. **(b-d)** ITC measurements of Nbs binding to the NabFab. The K_d values for Nb725_4 and the Anti-Fab Nb binding to NabFab were presented as the mean ± sem and the number of tests = 2. *N*, the binding stoichiometry number. The interaction of the parent Nb725 to the NabFab was too weak to be fit accurately. **(e)** Isolation of the MelB$_{St}$/Nb725_4/NabFab/anti-Fab Nb complex. MelB$_{St}$ proteins were reconstituted into the

lipid nanodiscs with the membrane scaffold protein 1E3D1 (MSP1E3D1). Sample containing $MelB_{St}$ (black curve), $MelB_{St}$ with Nb725_4 (blue curve), or with Nb725_4, NabFab, and anti-Fab Nb (red curve) were prepared prior to analysis by gel filtration chromatography in a combination of the SDS-15%PAGE. The mix-2 sample, $MelB_{St}$ with Nb725_4, blue color; mix-4 sample, $MelB_{St}$ with Nb725_4, NabFab and anti-Fab Nb, red color. The mixture of each protein in the loading buffer does not contain a reducing agent.

sFig. 2

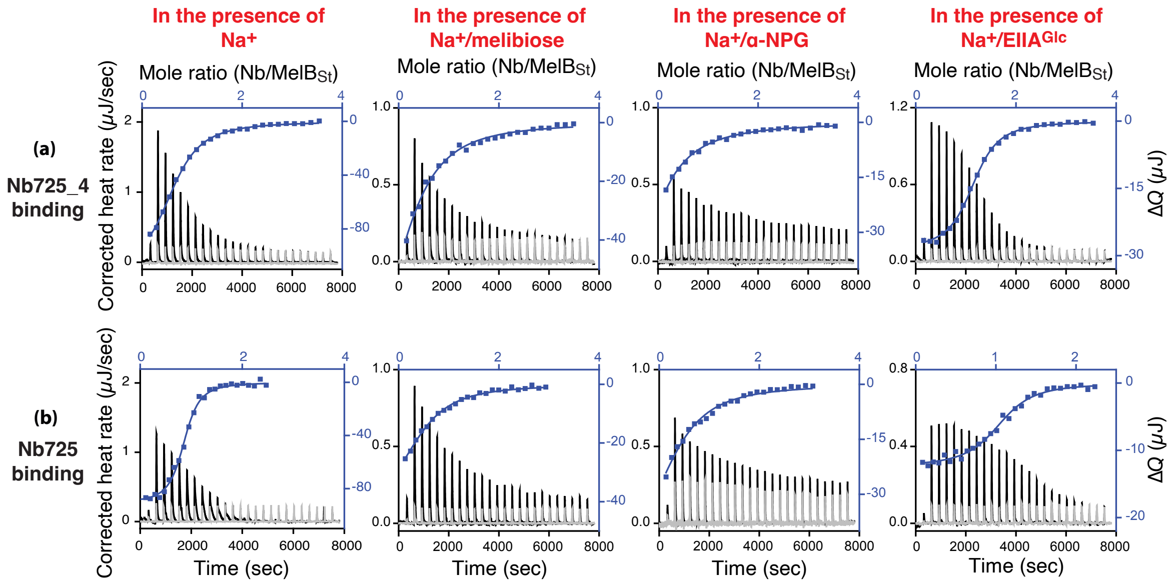

sFig. 2. Effects of substrate/ligand binding on the Nbs binding. All ITC binding measurements at 25 °C and curve fitting were described in the Methods. The purified Nb725_4, Nb725, $MelB_{St}$, and $EIIA^{Glc}$ protein samples were dialyzed against an identical buffer containing 100 mM NaCl. $MelB_{St}$ at a concentration of 35 μM was placed in the sample cells and titrated with Nb725_4 or Nb725, respectively. The thermograms of each titration (black) and the corresponding control by titrating Nbs into the protein-free buffer (light gray) were plotted by the bottom/left (x/y) axes. The binding isotherm and fitting using a one-site independent-binding model presented by top/right (x/y) axes. **(a)** Binding of Nb725_4 to the Na^+-bound $MelB_{St}$ in the absence or presence of melibiose (80 mM), α-NPG (5 mM), or $EIIA^{Glc}$ (2:1 ratio to $MelB_{St}$). **(b)** Binding of Nb725 to the Na^+-bound $MelB_{St}$ in the absence or presence of melibiose (80 mM), α-NPG (5 mM), or $EIIA^{Glc}$ (2:1 molar ratio to $MelB_{St}$).

sFig. 3

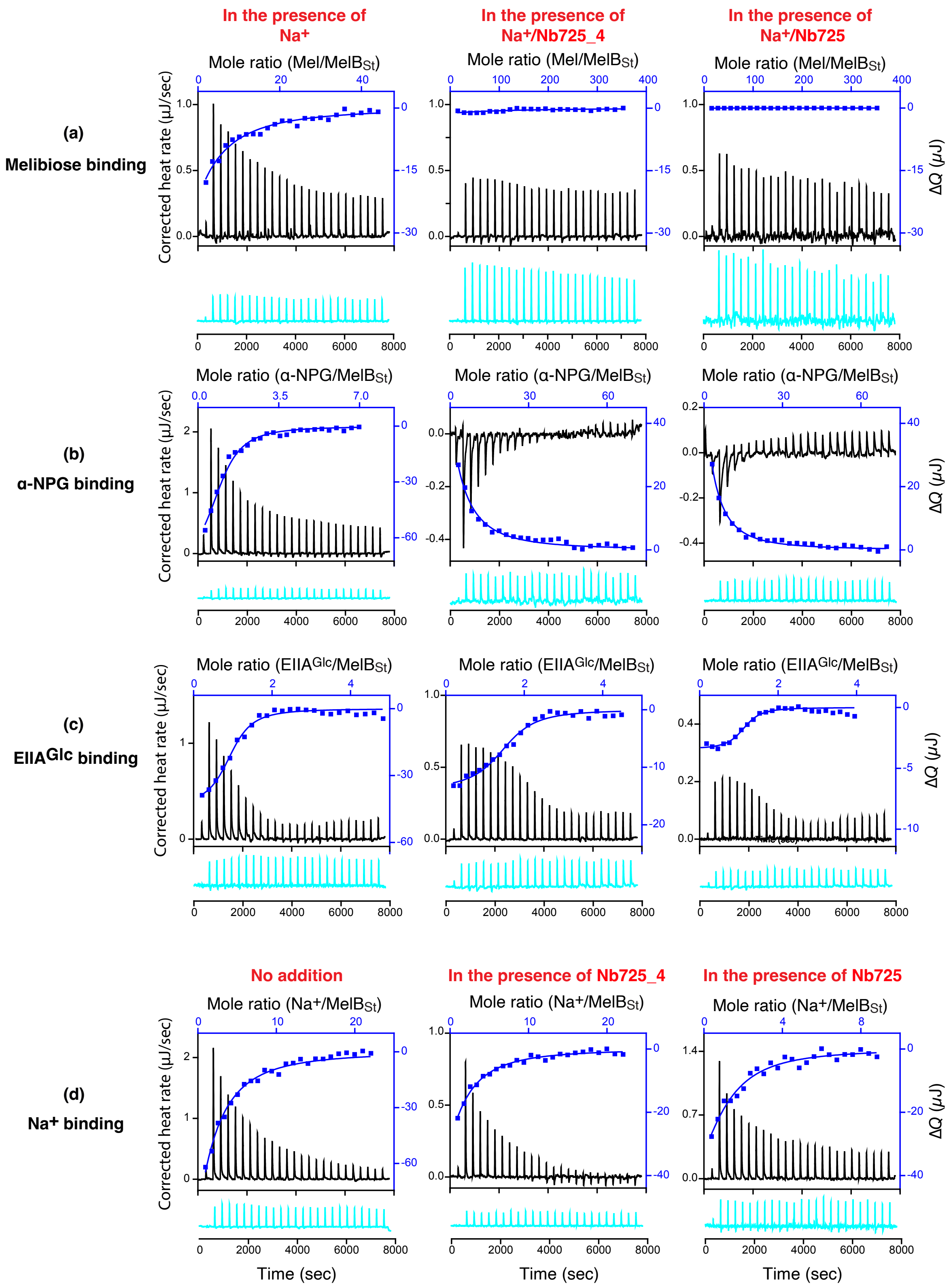

In the presence of Na+
In the presence of Na+/Nb725_4
In the presence of Na+/Nb725
(a)
Melibiose binding
Mole ratio (Mel/MelBSt)
(b)
α-NPG binding
Mole ratio (α-NPG/MelBSt)
(c)
EIIAGlc binding
Mole ratio (EIIAGlc/MelBSt)
No addition
In the presence of Nb725_4
In the presence of Nb725
(d)
Na+ binding
Mole ratio (Na+/MelBSt)
Corrected heat rate (μJ/sec)
ΔQ (μJ)
Time (sec)

sFig. 3. Nb effects on substrate/ligand binding. All ITC binding measurements at 25 °C and curve fitting were described in the Methods. The binding measurements of melibiose, α-NPG and $EIIA^{Glc}$ were conducted in buffer, 20 mM Tris-HCl, pH 7.5, 100 mM NaCl, 0.01% DDM, and 10% glycerol. The Na^+ binding measurements were conducted in 20 mM Tris-HCl, pH 7.5, 100 mM choline-Cl, 0.01% DDM, and 10% glycerol as described in (Hariharan, 2017). All proteins and ligand solutions were buffer-matched. $MelB_{St}$ complexed with Nb725_4 or Nb725 were prepared by mixing them at a 1:2 molar ratio. The thermograms of each titration (black) were plotted by bottom/left (x/y) axes and the binding isotherm and fitting using one-site independent-binding model presented by top/right (x/y) axes. The corresponding controls by titrating each ligand into the protein-free buffer (cyan) were presented below at an identical scale. **(a)** Titration of melibiose to $MelB_{St}$ or a given $MelB_{St}$/Nb complex at 80 µM for $MelB_{St}$. **(b)** Titration of α-NPG binding to $MelB_{St}$ a given $MelB_{St}$/Nb complex at 50 µM for $MelB_{St}$. **(c)** Titration of Na^+ to $MelB_{St}$ or a given $MelB_{St}$/Nb complex at 80 µM for $MelB_{St}$**.** **(d)** Titration of $EIIA^{Glc}$ to $MelB_{St}$ or a given $MelB_{St}$/Nb complex at 50 µM for $MelB_{St}$. For the titration into the $MelB_{St}$/Nb complexes, melibiose and α-NPG in the assay solutions were increased from 10 mM to 80 mM or 1 mM to 10 mM. The Na^+ at 3 mM and $EIIA^{Glc}$ at 0.5 mM were applied for titrations.

sFig. 4a

Overall strategy for single-particle resonstruction

14,094 micrographs collected
13,649 curated

(a) Initial 3D reconstruction

Template pick from 13,649 micrographs

2D Classification
Heterogeneous Refinement
3D Ab-inito Reconstruction
Non-uniformed Refinemnt

41,264-particle set

GSFSC resolution = 3.49 Å

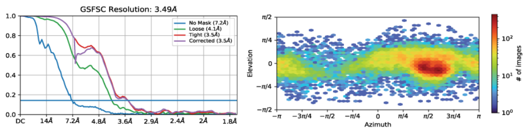

(b) Map improvement

Template pick from 13,649 micrographs

2D Classification
Heterogeneous Refinement
Local Refinement

Combinded/removed duplicates
Heterogeneous Refinement

188,566-particle set

GSFSC resolution = 3.58 Å

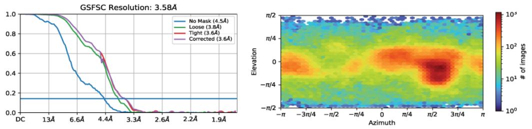

Re-cleaned particles focusing top/bottom views
from the rejected 2D classes

2D Classfication
Heterogeneous Refinement
Local Refinement

Combinded/removed duplicates
Heterogeneous Refinement
Local Refinement

196,254-particle set

GSFSC resolution = 3.39 Å

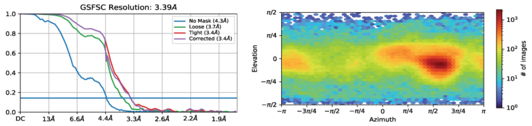

(c) Tilted data collection

8,716 micrographs tilted collection at 30 °
7,129 curated

Template pick from 7,129 micrographs

2D Classfication
Heterogeneous Refinement
Local Refinement

182,101 particles selected

Combinded
Heterogeneous Refinement
Local Refinement

378,355-particle set

GSFSC resolution = 3.40 Å

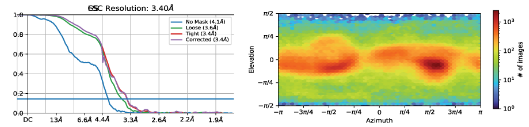

3D Classfication
Heterogeneous Refinement
Local Refinement

296,925-particle set

GSFSC resolution = 3.37 Å

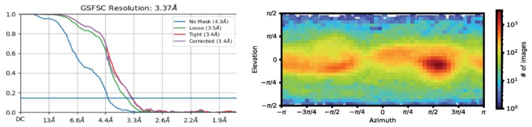

sFig. 4b

Initial 3D reconstruction

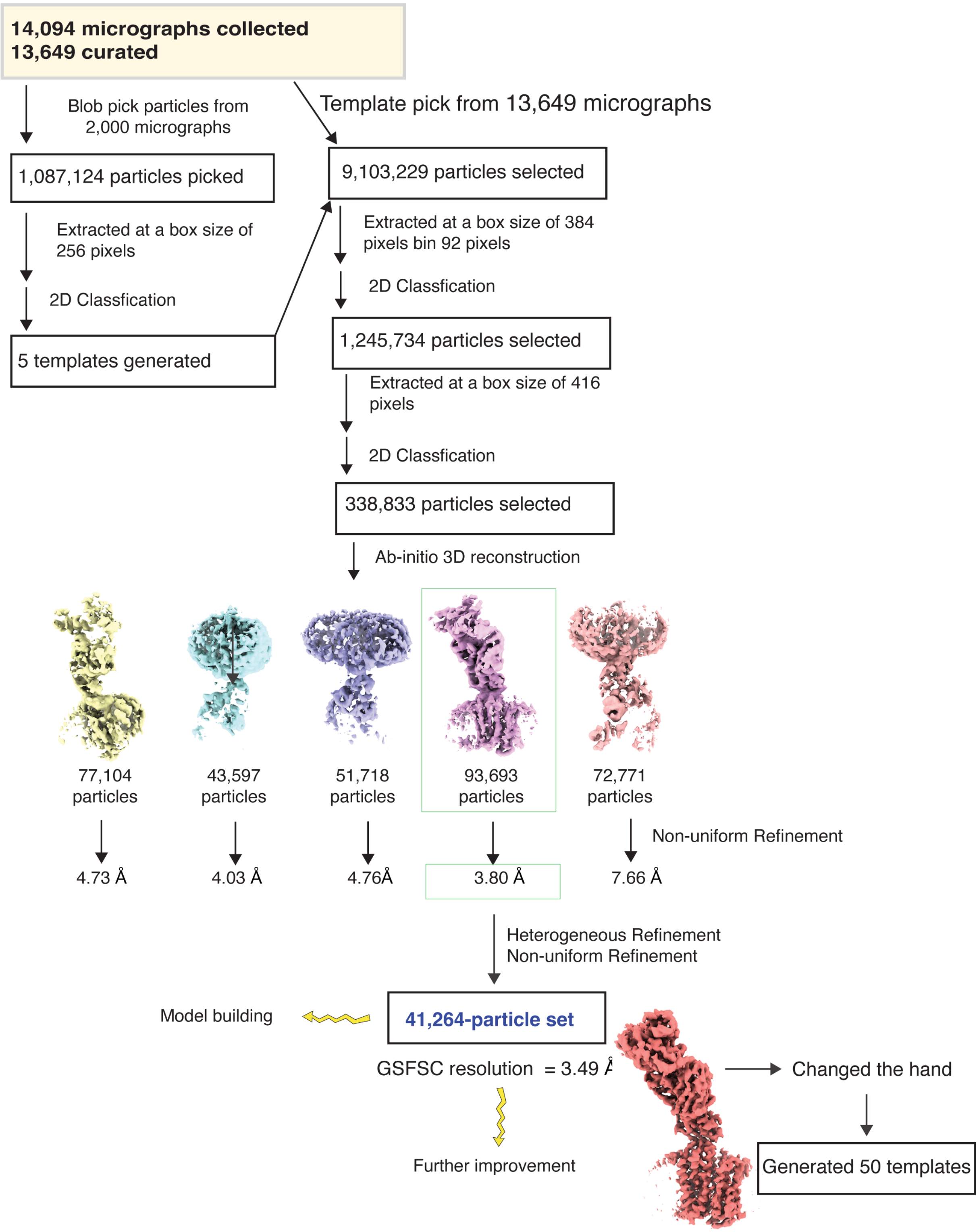

sFig. 4c

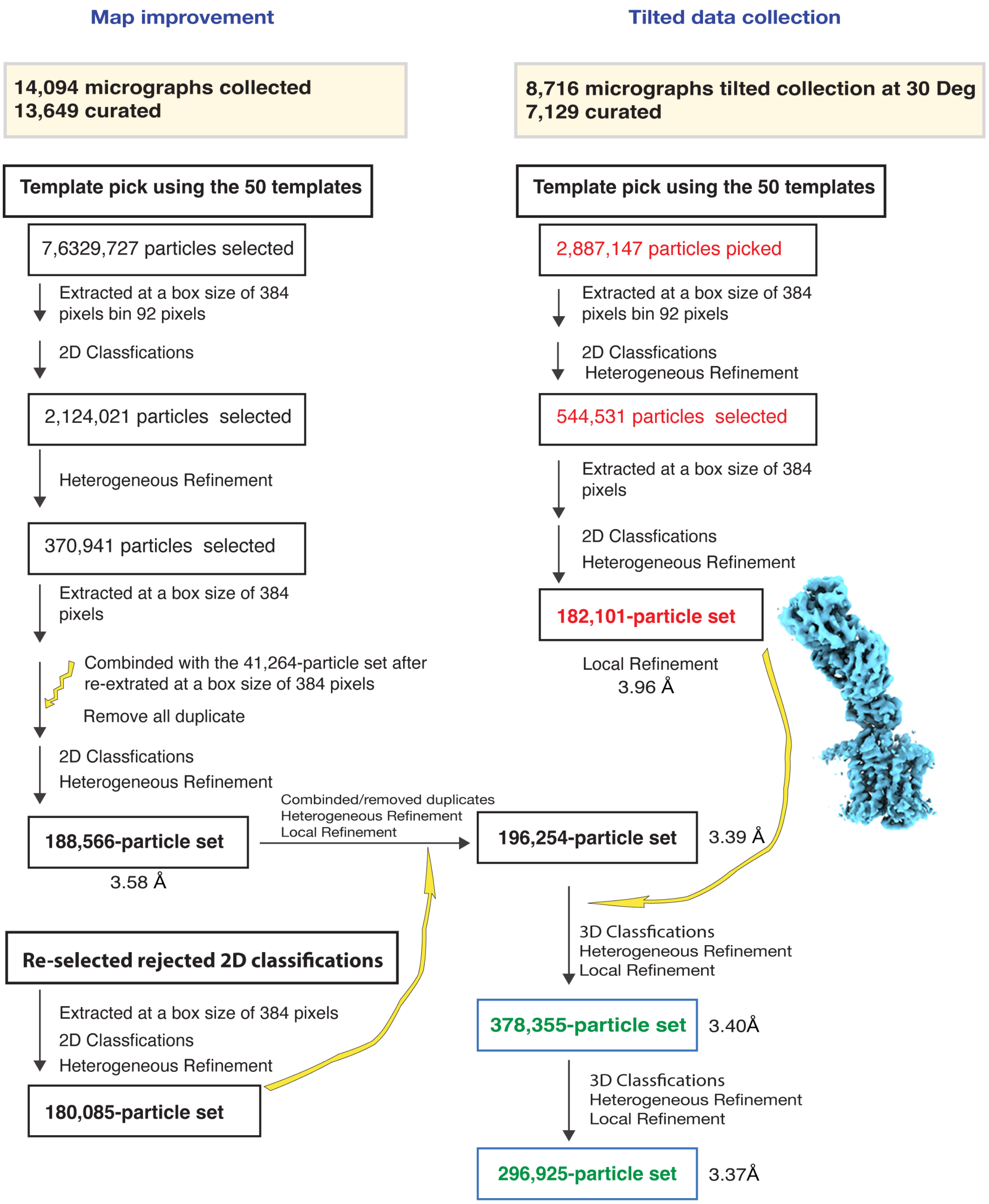

sFig. 4. CryoEM data process. The strategy for the reconstruction and refinement was outlined and the details were presented in panels b-c.

sFig. 5

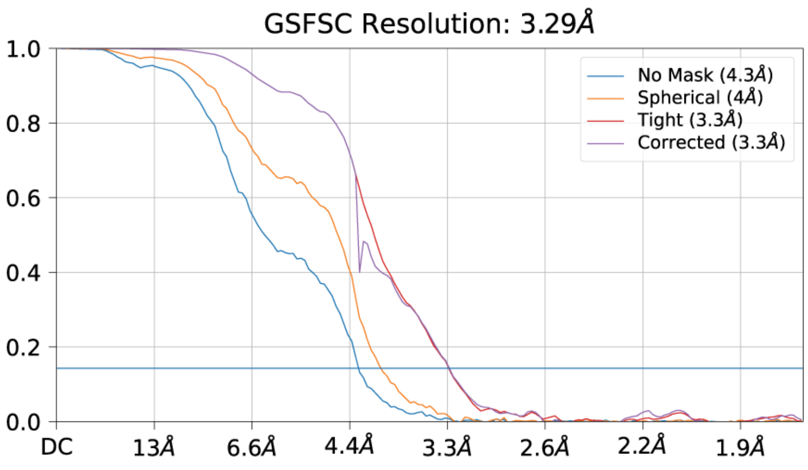

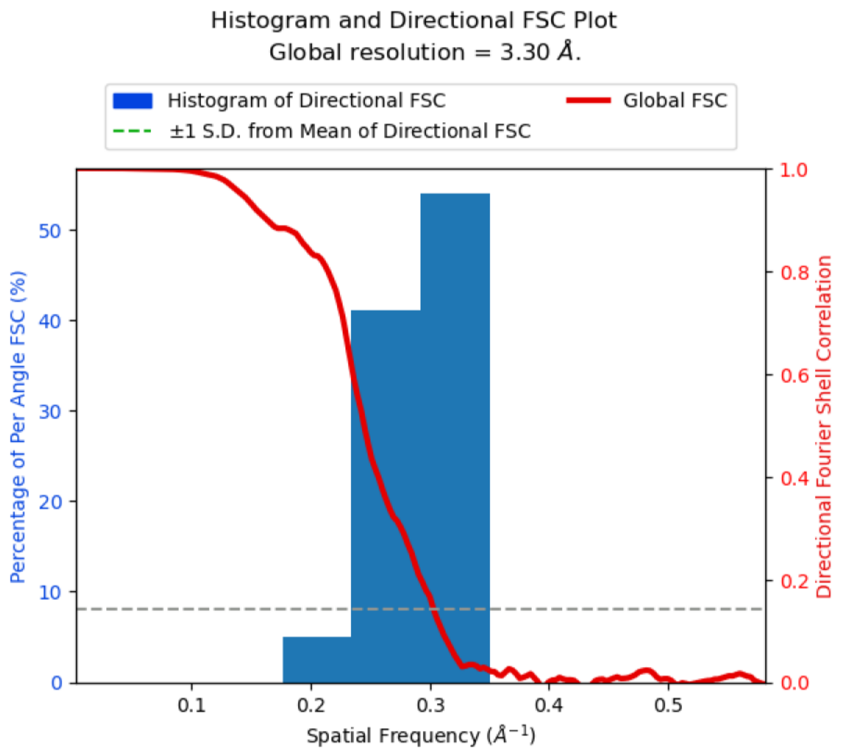

sFig. 5. GSFSC resolution and 3dFSC. The resolution of map and particle orientation distribution was assessed by cryoSPARC program using default setting parameters.

sFig. 6

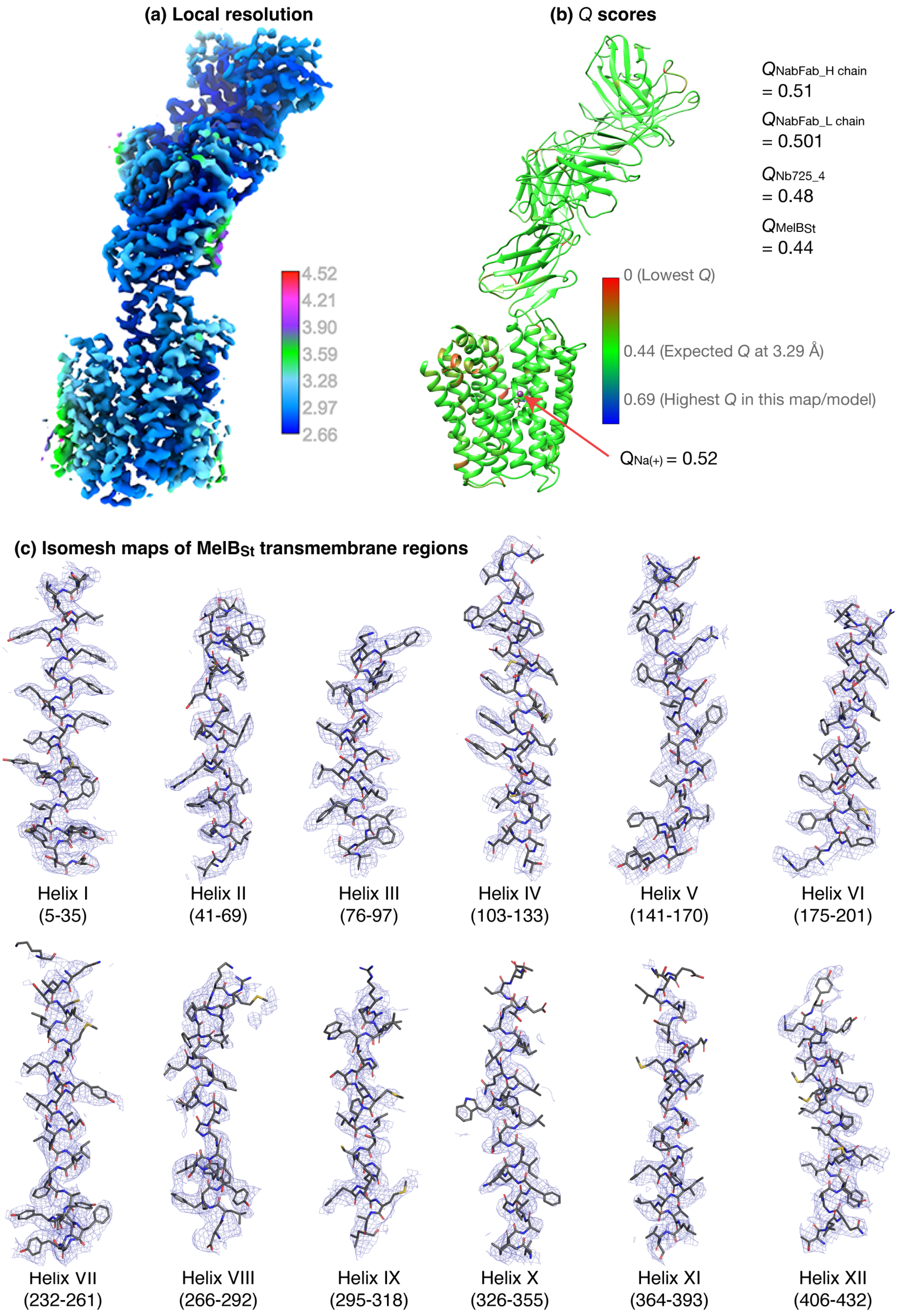

(a) Local resolution
4.52
4.21
3.90
3.59
3.28
2.97
2.66
(b) Q scores
$Q_{NabFab\_H\ chain}$ = 0.51
$Q_{NabFab\_L\ chain}$ = 0.501
Q_{Nb725_4} = 0.48
Q_{MelBSt} = 0.44
0 (Lowest Q)
0.44 (Expected Q at 3.29 Å)
0.69 (Highest Q in this map/model)
$Q_{Na(+)}$ = 0.52
(c) Isomesh maps of $MelB_{St}$ transmembrane regions
Helix I (5-35)
Helix II (41-69)
Helix III (76-97)
Helix IV (103-133)
Helix V (141-170)
Helix VI (175-201)
Helix VII (232-261)
Helix VIII (266-292)
Helix IX (295-318)
Helix X (326-355)
Helix XI (364-393)
Helix XII (406-432)

sFig. 6. Evaluation of map and models. (a) The local resolution. The map half_A and half_B files produced by the cryoSPARC Local Refinement program were used to calculate the Local Resolution Map by Phenix and displayed by UCSF ChimeraX using the defined color key. **(b)** *Q* scores. The auto-sharpened map generated by Phenix under the default setting was used as the main map for the model building and also for *Q*-score calculations by Map*Q* program in UCSF Chimera against the final structure at a sigma level of 0.4. The results of the *Q* scores for each residue were color-painted on the structure using the given color key. The bound Na^+ was shown as a sphere. The expected score at a resolution of 3.29 Å is 0.44. **(c)** Density mapped on the $MelB_{St}$ transmembrane helices. The auto-sharped map by Phenix was used to calculate the isomesh maps for each transmembrane helix at a sigma level of 10 and carving of 1.8.

sFig. 7

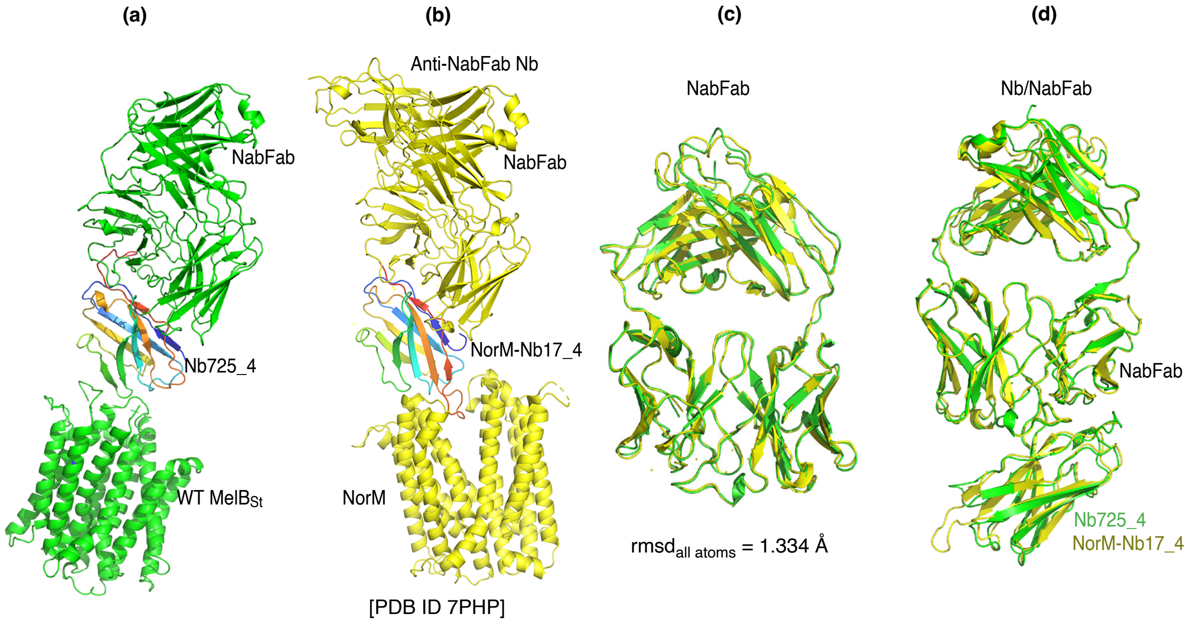

sFig. 7. NabFab comparison. (**a & b**) NorM complexed with anti-NorM Nb17_4/NabFab/anti-NabFab Nb [PDB ID 7PHP] was superimposed with MelB$_{St}$ complexed with Nb725-4 and NabFab based on the H chains of NabFab in the two structures. Nb725-4 and NorM-Nb17_4 were colored in the rainbow. (**c**) The NabFab in the NorM complex [PDB ID 7PHP] was superimposed with that in MelB$_{St}$ complex. (**d**) The NabFab/Nb in the NorM complex was superimposed with that in MelB$_{St}$ complex based on their H chains. Green, MelB$_{St}$ complex; yellow, NorM complex.

sFig. 8

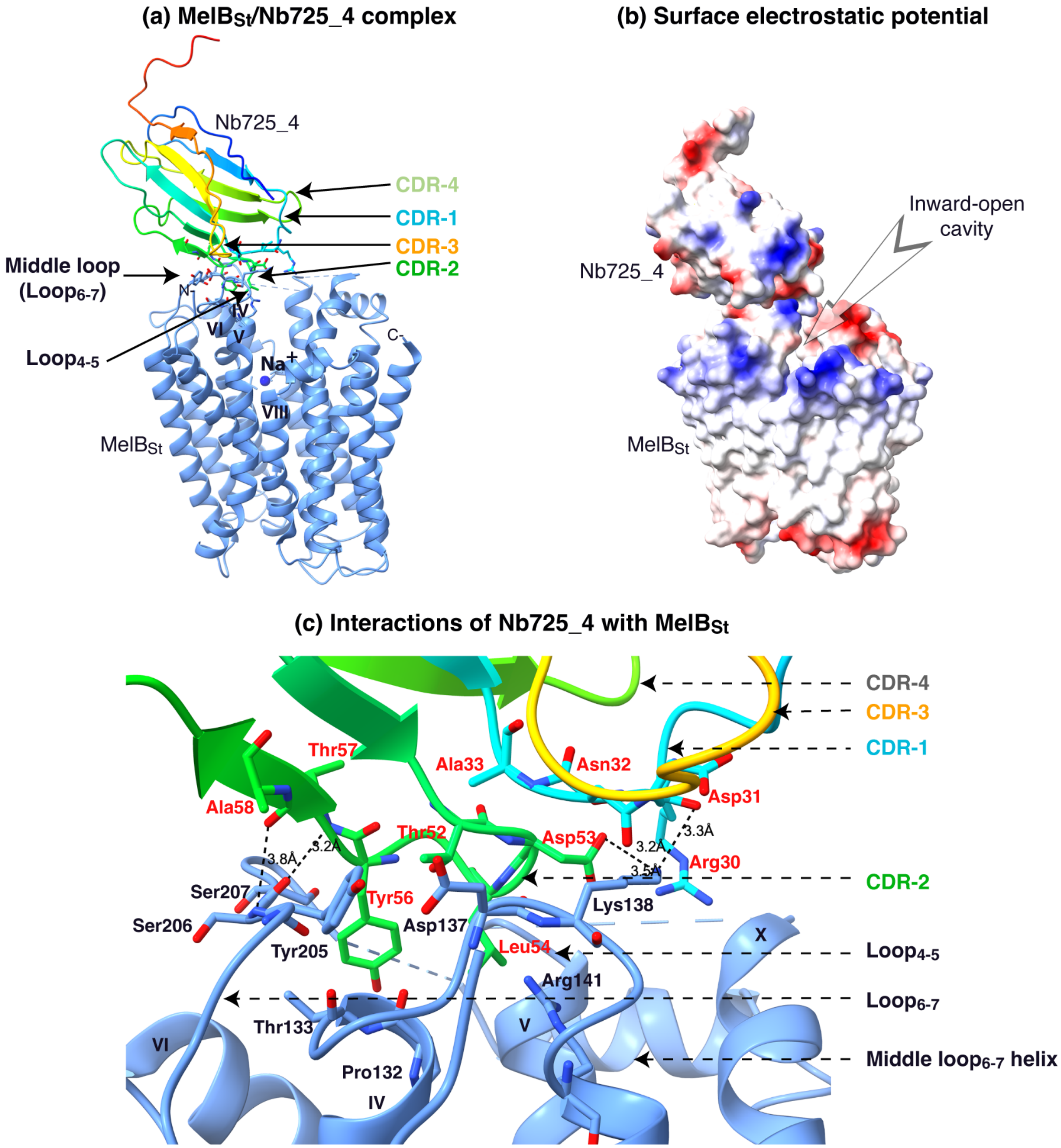

sFig. 8. Interactions of Nb725m_4 and MelB$_{St}$. (a) Nb725_4 bound to the N-terminal domain of MelB$_{St}$. The contact between Nb CDR-1 and CDR-2 and MelB$_{St}$ loop$_{4\text{-}5}$, and loop$_{6\text{-}7}$ contributed to the major interactions. **(b)** The surface electro potential map was generated in the UCSF Chimera X program. The inward-open cavity is indicated. **(c)** The binding interface. All residues with a buried solvent-accessible surface area >15 Å^2 or polar interaction at a distance <3.5 Å were selected by the UCSF Chimera X program were highlighted in sticks. The salt-bridging and hydrogen-bonding interactions were highlighted by the dashed line. The Nb CDRs and epitope are indicated.

sFig. 9

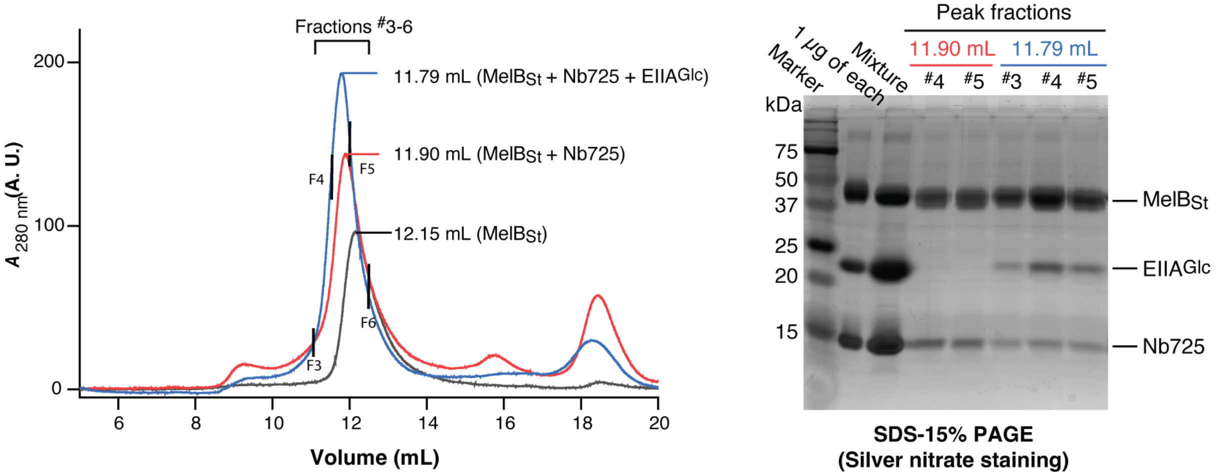

sFig. 9. Complex of MelB$_{St}$ with Nb725 and EIIA^{Glc}. Samples containing MelB$_{St}$ (black curve), MelB$_{St}$ with Nb725 (red curve), or with Nb725 and EIIA^{Glc} (blue curve), were prepared and analyzed by gel filtration chromatography in a combination of the SDS-15%PAGE stained by silver nitrate. Mixture, the solution was prepared prior to the gel filtration chromatography containing all three proteins. 1 µg of each protein was loaded as the control.

sFig. 10

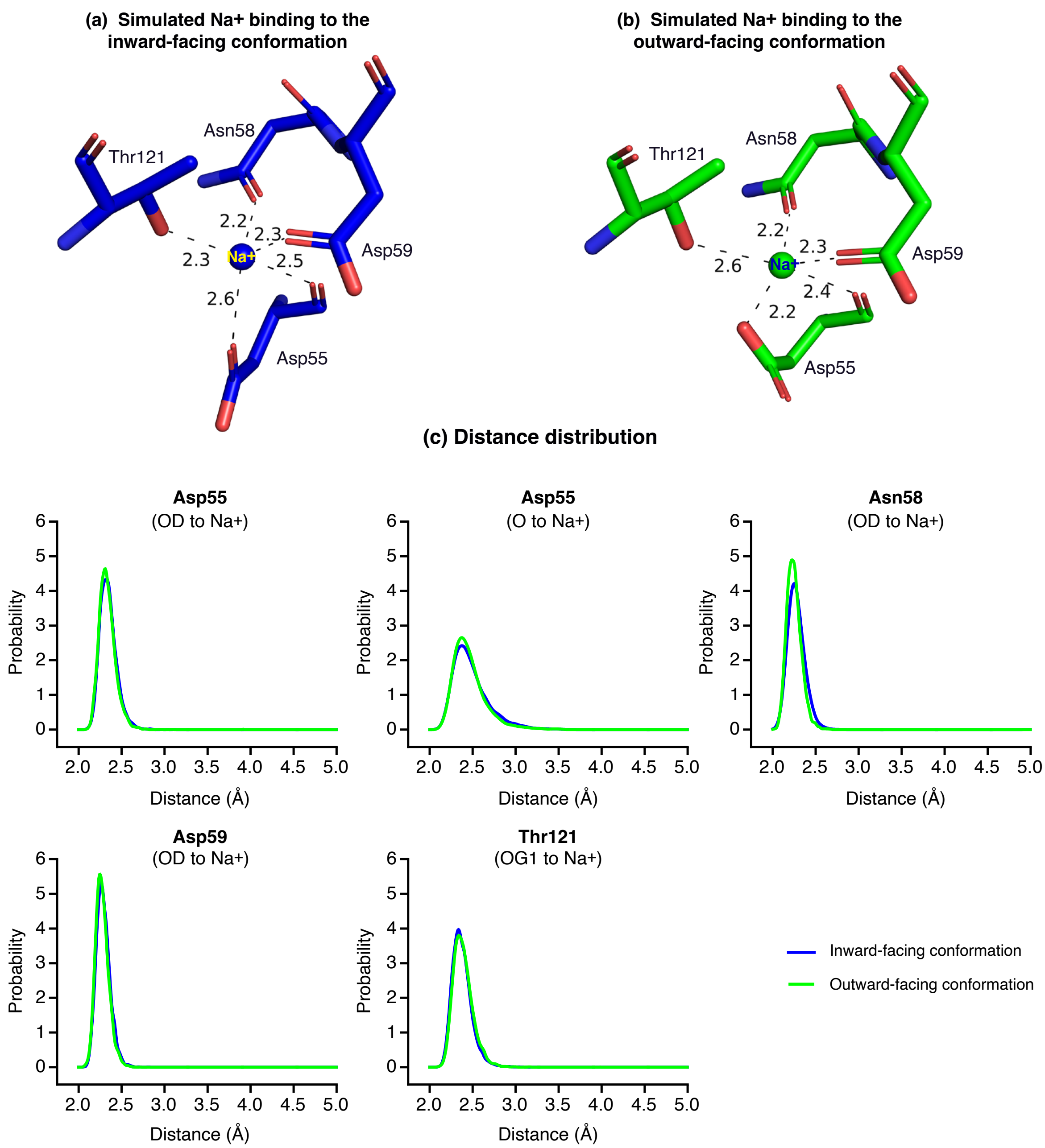

sFig. 10. MD simulations of the Na^+ binding at both inward- and outward-facing states. The equilibrated structures of Na^+-binding site for the inward- and outward-facing $MelB_{St}$ in the absence of melibiose. Distances between Na^+ and nearby residues' coordinating atoms are labeled in text and dashed lines. **(a)** Na^+ binding in the inward-facing conformation. **(b)** Na^+ binding in the outward-facing conformation. **(c)** Probability distribution of the distance between the bound Na^+ to all ligands. Blue, inward-facing conformation; green, outward-facing conformation.

sFig. 11

Outward-facing X-ray crystal structure

[PDB ID 7L17; 3.05 Å]

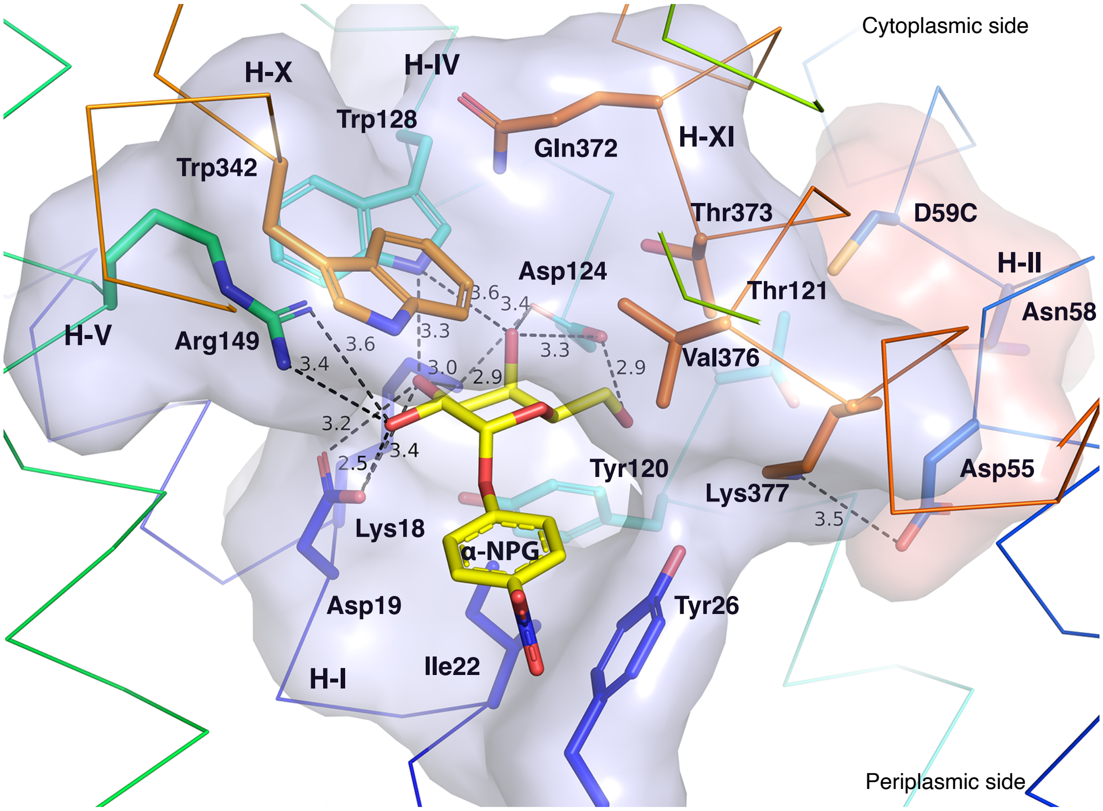

sFig. 11. Galactose-binding pocket in the outward-facing crystal structure [PDB ID 7L17]. All polar interactions between the bound α-NPG and D59C $MelB_{St}$ were indicated by dash lines and the pocket was shown in surface representation in blue. The Na^+-binding residues were highlighted by sticks and surface representation in pink. The cytoplasmic and periplasmic sides were indicated. Eight N-terminal residues (Lys18, Asp19, Ile22, Tyr26, Tyr120, Asp124, Trp128, and Arg149), especially the four charged residues, form the multiple polar interactions with one surface of the galactopyranosyl moiety. Five C-terminal residues (Trp342, Gln372, Thr373, Val376, and Lys377) form a non-specific barrier without a strong polar interaction

sFig. 12

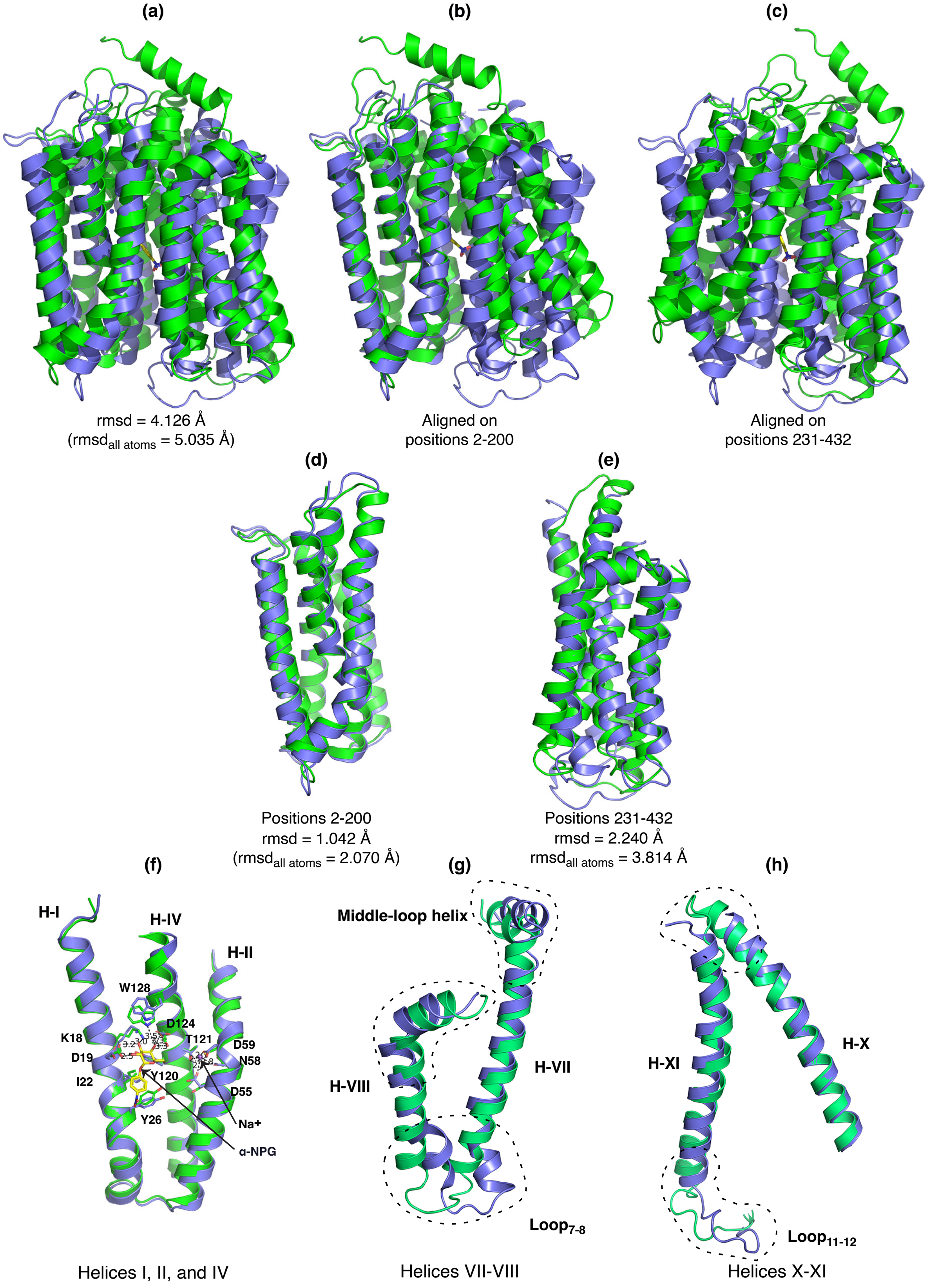
(a)
(b)
(c)
rmsd = 4.126 Å
(rmsd$_{all\ atoms}$ = 5.035 Å)
Aligned on
positions 2-200
Aligned on
positions 231-432
(d)
(e)
Positions 2-200
rmsd = 1.042 Å
(rmsd$_{all\ atoms}$ = 2.070 Å)
Positions 231-432
rmsd = 2.240 Å
rmsd$_{all\ atoms}$ = 3.814 Å
(f)
(g)
(h)
H-I
H-IV
H-II
W128
D124
K18
T121
D59
D19
N58
I22
Y120
D55
Y26
Na+
α-NPG
Middle-loop helix
H-VIII
H-VII
Loop$_{7-8}$
H-XI
H-X
Loop$_{11-12}$
Helices I, II, and IV
Helices VII-VIII
Helices X-XI

sFig. 12. Alignment. The alignment of the sugar-bound outward-facing structure (7L17, green) with the Na^+-bound inward-facing structure (blue) were carried out in Pymol or Coot programs. The rmsd values reported from Pymol program were either based on default settings with outlier rejection or using all atoms after removing unmatched residues. **(a)** Superposition of both structures. (**b**) Full-length alignment based on positions 2-200. **(c)** Full-length alignment based on positions 231-332. (**d)** Focused alignment of positions 2-200. **(e)** Focused alignment of positions 231-432. **(f)** Helices I, II, and IV isolated from the focused alignment of positions 2-300 in *d*. The major binding residues for Na^+ and α-NPG were highlighted. **(g)** Helices VII-VIII and the extended loops isolated from the focused alignment of positions 219-432. **(h)** Helices X-XI and the extended loops isolated from the focused alignment of positions 219-432.

sFig. 13

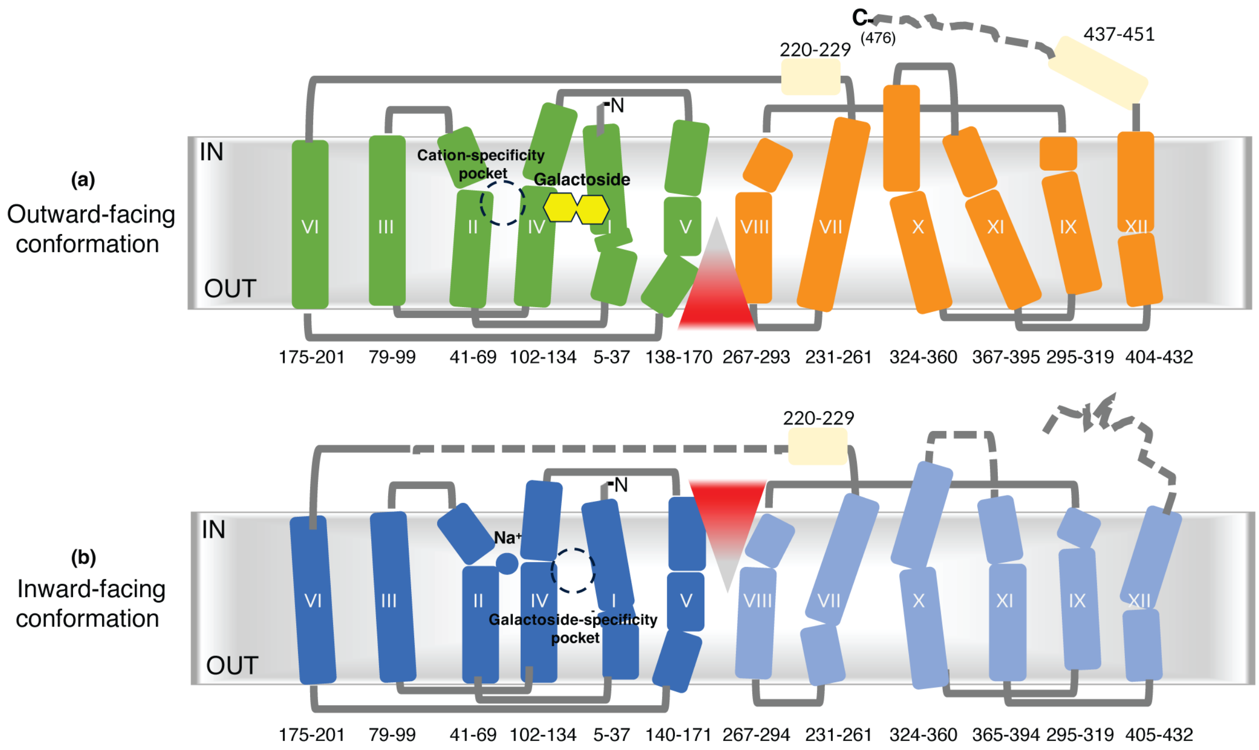

sFig. 13. Membrane topology. (a) The sugar-bound outward-facing structure (PDB ID 7L17). **(b)** The Na^+-bound inward-facing structure. The full-length $MelB_{St}$ is illustrated by transmembrane topology based on both structures. The residue positions for each helix are indicated. The N- and C-terminal transmembrane helices are colored green and orange for the outward-facing conformation, respectively, as well as blue and light blue for the inward-facing conformation, respectively. The peripheral helices are colored in light yellow. Blue ball, Na^+; yellow hexagons, galactoside. The red triangle indicates the solvent-access paths. Grey lines, loops; dashed lines, un-resoled loops. Broken circles, either cation-specificity pocket or sugar-specificity pocket.

sFig. 14

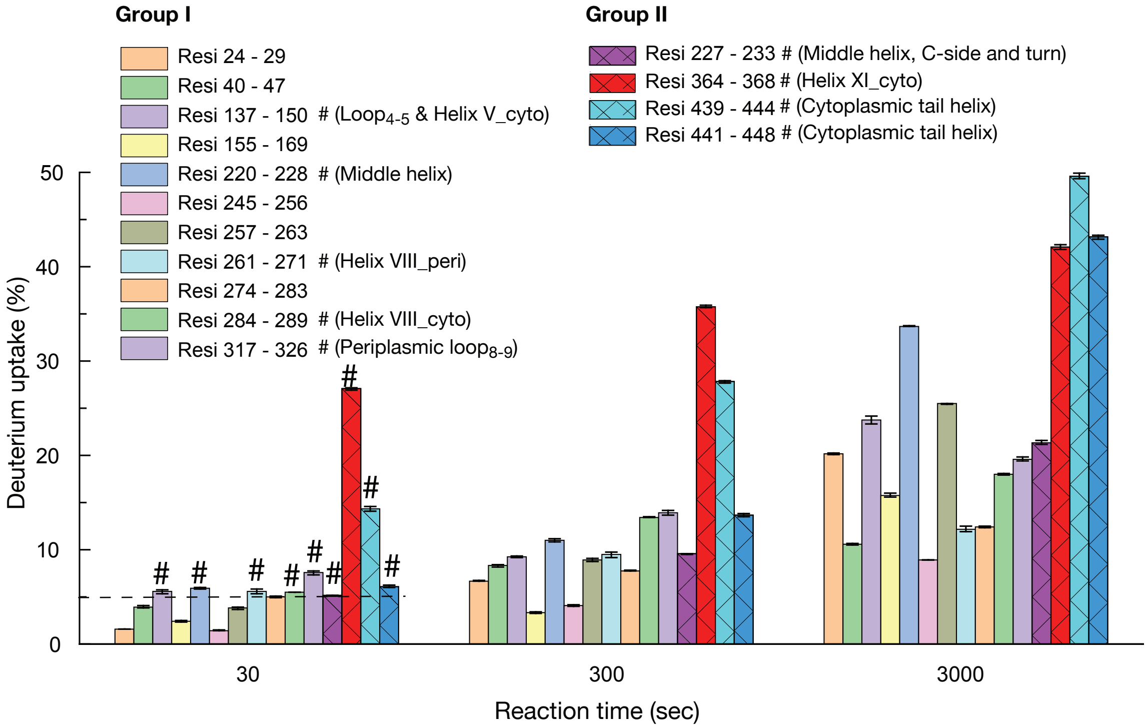

sFig. 14. Histograms of deuterium uptake time courses. The deuterium uptake time courses of the groups I&II presented in the Fig. 7 were replotted in the histogram. Error bar, sem; test number, 3.
