# Supplementary material for: Mobile barrier mechanisms for Na+-coupled symport in an MFS sugar transporter
Source: bioRxiv. 2023 Nov 25:2023.09.18.558283. Originally published 2023 Sep 18. Preprint. [Version 2] doi: 10.1101/2023.09.18.558283 (PMC10542114; doi:10.1101/2023.09.18.558283)

Extended Fig. 1

Deuterium uptake time-course of all peptides with statastically significant changes

(Coverage of residue positions 2 - 263)

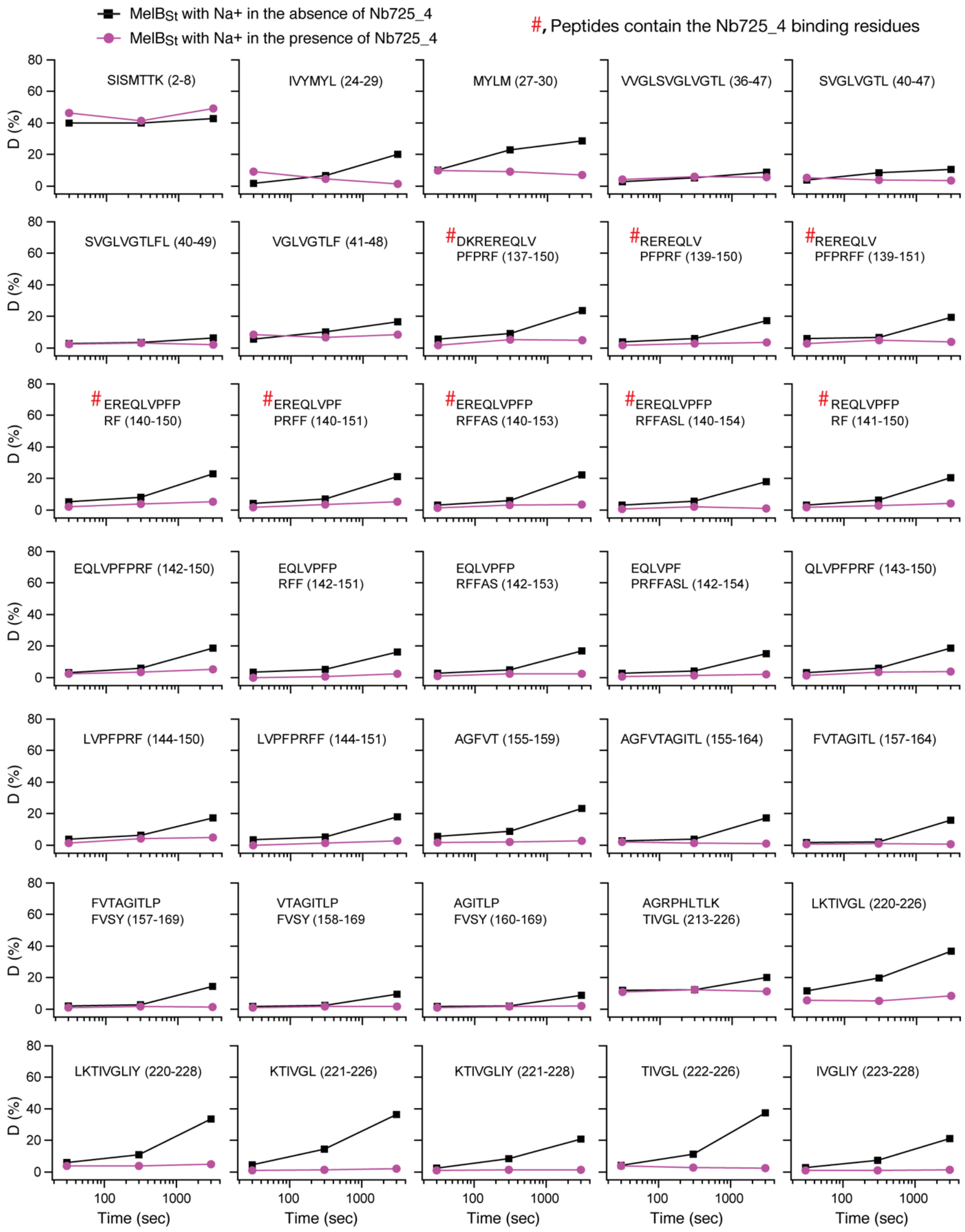

(Coverage of residue positions 261 - 475)

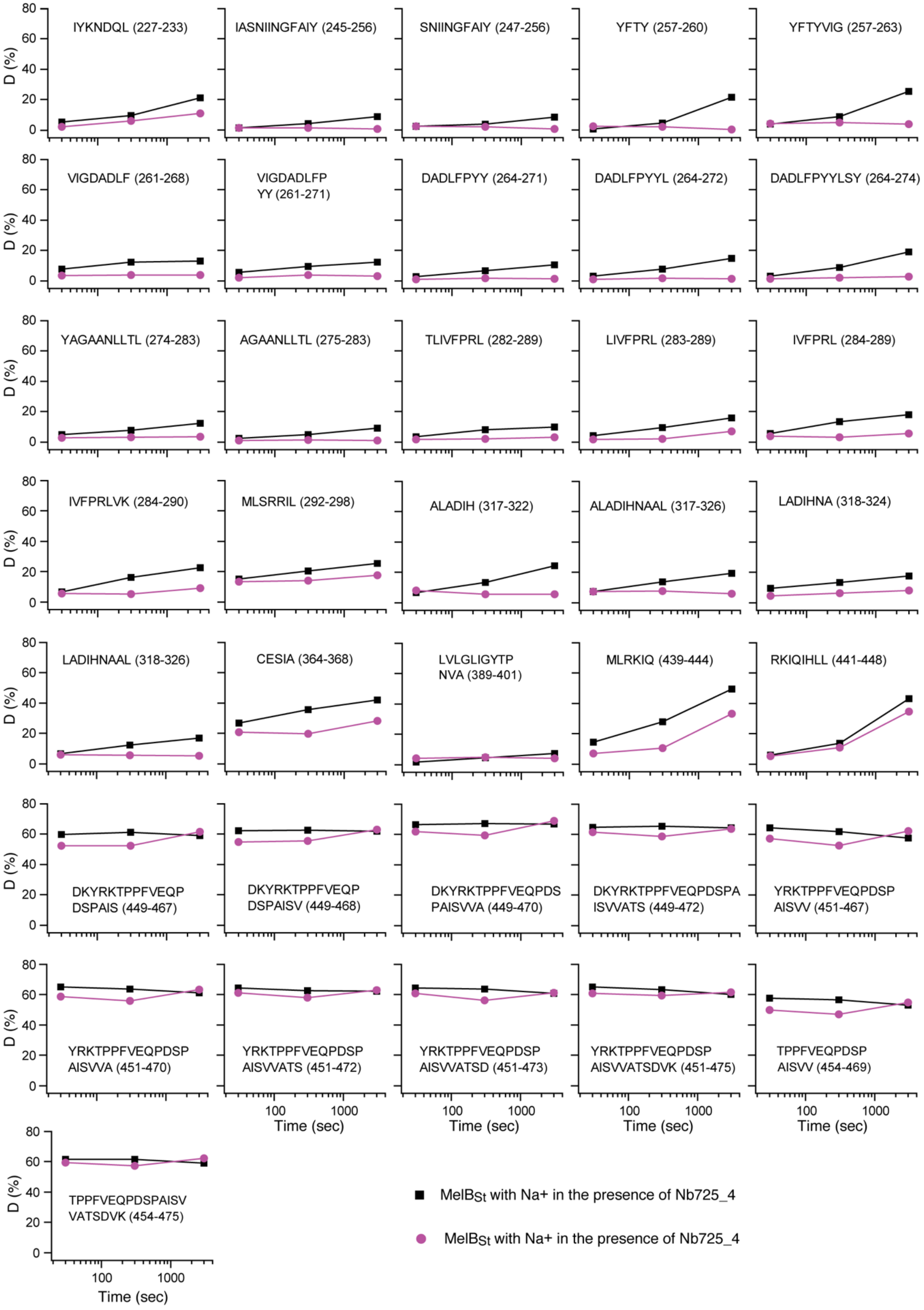

Supplement: Supplement 2 [file media-2.pdf]
